# Quantitative Measurements of LRRK2 in Human Cerebrospinal Fluid Demonstrates Increased Levels in G2019S Patients

**DOI:** 10.3389/fnins.2020.00526

**Published:** 2020-05-25

**Authors:** Omar S. Mabrouk, Siwei Chen, Amanda L. Edwards, Minhua Yang, Warren D. Hirst, Danielle L. Graham

**Affiliations:** ^1^Clinical Sciences, Biomarkers, Biogen, Cambridge, MA, United States; ^2^Biostatistics, Biogen, Cambridge, MA, United States; ^3^Neurodegeneration Research Unit, Biogen, Cambridge, MA, United States

**Keywords:** LRRK2, Parkinson’s disease, G2019S, cerebrospinal fluid, exosome, biomarkers, mass spectrometry, SISCAPA

## Abstract

Leucine-rich repeat kinase 2 (LRRK2) mutations are among the most significant genetic risk factors for developing late onset Parkinson’s disease (PD). To understand whether a therapeutic can modulate LRRK2 levels as a potential disease modifying strategy, it is important to have methods in place to measure the protein with high sensitivity and specificity. To date, LRRK2 measurements in cerebrospinal fluid (CSF) have used extracellular vesicle enrichment via differential ultracentrifugation and western blot detection. Our goal was to develop a methodology which could be deployed in a clinical trial, therefore throughput, robustness and sensitivity were critical. To this end, we developed a Stable Isotope Standard Capture by Anti-peptide Antibody (SISCAPA) assay which is capable of detecting LRRK2 from 1 ml of human CSF. The assay uses a commercially available LRRK2 monoclonal antibody (N241A/34) and does not require extracellular vesicle enrichment steps. The assay includes stable isotope peptide addition which allows for absolute quantitation of LRRK2 protein. We determined that the assay performed adequately for CSF measurements and that blood contamination from traumatic lumbar puncture does not pose a serious analytical challenge. We then applied this technique to 106 CSF samples from the MJFF LRRK2 Cohort Consortium which includes healthy controls, sporadic PD patients and LRRK2 mutation carriers with and without PD. Of the 105 samples that had detectable LRRK2 signal, we found that the PD group with the G2019S LRRK2 mutation had significantly higher CSF LRRK2 levels compared to all other groups. We also found that CSF LRRK2 increased with the age of the participant. Taken together, this work represents a step forward in our ability to measure LRRK2 in a challenging matrix like CSF which has implications for current and future LRRK2 therapeutic clinical trials.

## Introduction

Leucine-rich Repeat Kinase 2 (LRRK2) is a large (280 kDa) GTPase/kinase involved in intracellular vesicle dynamics, autophagy and inflammation processes (Fraser et al., 2013; Arranz et al., 2015; Wallings and Tansey, 2019). Given that LRRK2 mutations are among the most frequent genetic cause of Parkinson’s disease (PD), it has become an attractive therapeutic target with at least 2 ongoing interventional clinical trials at the time of this publication^1^. To advance LRRK2 therapeutic development, measuring LRRK2 in cerebrospinal fluid (CSF) as an indirect central nervous system (CNS) target engagement and/or patient stratification biomarker would be advantageous. For instance, CSF LRRK2 concentrations could serve as a patient enrichment tool and/or a pharmacodynamic endpoint if a therapy aims to modulate aberrant CNS levels, e.g., with an antisense oligonucleotide (ASO) or gene therapy approach. Importantly, an outstanding question in the field is whether PD patients with LRRK2 mutations (such as G2019S) have altered expression of total or phosphorylated LRRK2 in brain and CSF. To date, CSF LRRK2 detection has proven technically challenging. This has led the Michael J Fox Foundation (MJFF) to sponsor the LRRK2 Detection Consortium which is an industry/academia initiative aimed at promoting the development of technologies enabling LRRK2 detection in different matrices including PBMCs, urine and CSF.

LRRK2 in human CSF has been successfully measured using extracellular vesicle enrichment strategies (Fraser et al., 2013, 2016; Wang and West, 2019). Differential ultracentrifugation has been the preferred approach to isolate LRRK2-containing vesicles in CSF and urine followed by western blot (WB) for detection and quantitation (Fraser et al., 2013; Wang et al., 2017). Unfortunately, there are several difficulties in implementing this approach in a clinical trial setting. Processing CSF samples by ultracentrifugation may introduce variability which may be difficult to control. For instance, centrifuge type (swing bucket vs fixed angle), speed and performance consistency would be difficult to standardize across testing sites. Following CSF enrichment, a protein pellet may or may not be visible, therefore, resuspension of LRRK2 containing vesicles itself may be unreliable. WB analysis is considered low throughput, difficult to standardize, not sufficiently quantitative, and thus not amenable to clinical trials. Another important point to consider for a LRRK2 clinical endpoint is that CSF LRRK2 appears to vary greatly between subjects (Fraser et al., 2016; Wang et al., 2017) and therefore a clinical trial ready LRRK2 assay must have a wide dynamic range to capture biological variance.

An alternative to WB-based protein detection is enzyme-linked immunosorbent assay (ELISA) which is suited toward measuring proteins with far higher throughput. A number of high quality total LRRK2 mAbs and commercially available ELISA kits are available, however, to date there have been no reports demonstrating reliable CSF LRRK2 detection with these. Internal efforts from Biogen and other industry groups with support from the MJFF have developed ultrasensitive immunoassays (Singulex Erenna, Quanterix Simoa, MSD S-plex) to enable CSF LRRK2 detection (Padmanabhan et al., 2020). Despite single digit pg/ml sensitivity limits, and robust detection in rodent and primate tissues these assays were unable to reliably detect LRRK2 in human CSF (unpublished internal Biogen data).

Because of the limited applicability of ultracentrifugation/WB analyses and issues developing a high sensitivity ELISA based platform, our group sought to develop a SISCAPA (stable isotope standards and capture by anti-peptide antibody; Anderson et al., 2004) assay as an alternative approach This approach has the advantage of entirely denaturing biological samples with a protease such as trypsin. Following proteolysis, peptides (unique to protein of interest) are isolated by anti-peptide antibodies and then analyzed using high sensitivity mass spectrometry techniques. Isotopically labeled peptide (with identical amino acid sequence as detection peptide) is spiked into the sample to control for immunoprecipitation efficiency, LC-MS variability and is a convenient method for quantifying endogenous peptide (and thus protein).

We demonstrate that this approach enables consistent CSF LRRK2 detection from 1 ml of human CSF. Following assay qualification steps, we analyzed samples from the MJFF LRRK2 Cohort Consortium to understand whether LRRK2 levels were different between healthy controls and PD patients with and without G2019S LRRK2 mutations. The assay described here opens a new door into LRRK2 research where reliable quantitative measurements are needed to establish changes in the context of a LRRK2 therapeutic clinical trial.

## Materials and Methods

### SISCAPA Antibody Selection

According to epitope mapping data provided by MJFF (Table 1), several commercially available antibodies had epitopes that are contained within *in silico* determined tryptic peptides (i.e., do not contain a K or R within their sequence) including 8G10 (DEDGHFP), SIG-39840 (FPNEF) and N241A/34 (EGDLLVNPDQ). Of these three antibodies, preliminary experiments led us to select N241A/34 as a candidate anti-peptide antibody to isolate and measure the tryptic peptide AEEGDLLVNPDQPR (AA 1834–1847). This peptide was shown to be unique to LRRK2 protein (NIH, Standard Protein BLAST).

**TABLE 1:** Epitope mapping data showing the main epitopes of commercially available total LRRK2 monoclonal antibodies.

Antibody Sample	Species	LRRK2 Part	Spot Intensities	Main Epitope	Observations
MJFF1 (c5-8)	Rabbit	C-terminal (970–2527 aa)	High	LDLSANELRDI	None
MJFF2 (c41-2)	Rabbit	C-terminal (970–2527 aa)	High to very high	LSANELRDI	None
MJFF3 (c69-6)	Rabbit	C-terminal (970–2527 aa)	High	LDLSANELRDID	None
MJFF4 (c81-8)	Rabbit	C-terminal (970–2527 aa)	High	SANELRDID	None
MJFF5 (c68-7)	Rabbit	C-terminal (970–2527 aa)	High	LSANELRDI	None
SIG-39840	Mouse	C-terminal (970–2527 aa)	High to very high	FPNEF	Remarkable cross-reaction with peptides with the motifs FAGREEF and DELEF
N241A/34	Mouse	C-terminal (970–2527 aa)	High	EGDLLVNPDQ	None
N231B/34	Mouse	C-terminal (970–2527 aa)	Very weak	LKFPNEFD	Higher intensities with anti-rabbit Ab; cross-reaction with N-terminal motif DEDGHFP
UDD3	Rabbit	N-terminal (1–555 aa)	Very high	HEKI	Short consensus motif; cross-reaction with peptides with motif FFNILVLNEVHEFV
8G10	Mouse	N-terminal (1–555 aa)	High	DEDGHFP	None
N138/6	Mouse	N-terminal (1–555 aa)	Moderate	LNNVQEGKQI	None

*Reprinted with permission from the Michael J Fox Foundation for Parkinson’s disease research.*

### Post Immunoprecipitation Peptide Mapping

To test the hypothesis that N241A/34 could isolate the unique LRRK2 peptide that contains its epitope (i.e., AEEGDLLVNPDQPR), we conjugated Neuromab N241A/34 (Antibodies Inc, Davis CA) onto M-280 Tosylactivated Dynabeads (Thermo Fisher, Waltham, MA, United States) at a concentration of 1 ug N241A/34 per 1 μl of beads. We then digested 10 μg wild type recombinant LRRK2 (rLRRK2; Life Technologies) using 5 μg TPCK-treated trypsin (Worthington Biochemical, Lakewood, NJ, United States) at 40°C for 3 h shaking at 1400 RPM on an Eppendorf Thermomixer. Digestion was stopped by adding 5 μg of protease inhibitor AEBSF (Thermo Fisher, Waltham, MA, United States). Ten μl of N241A/34 bead solution (i.e., 10 μg of N241A/34) was added to the protein digest and immunoprecipitation was performed on an end over end Hula Mixer (Thermo Fisher, Waltham, MA, United States) for 1.5 h at 4°C. Sample was then placed on a DynaMag-2 magnetic tube holder (Thermo Fisher, Waltham, MA, United States) and beads were isolated from digest mixture. Beads were then washed using 1 ml of PBS + 0.05% Tween (PBST) on and end over end mixer for 1 min. PBST was removed and then washed twice using 1 ml PBS. Peptides were eluted off beads with 50 μl of H_2_0 + 0.1% formic acid and 5% acetonitrile (I). A Thermo Q Exactive Plus (Thermo Fisher, Waltham, MA, United States) orbitrap mass spectrometer was operating in data dependent acquisition (DDa) mode to search for the most abundant peptides eluted from beads. Full MS settings were 60,000 resolution, AGC target 1e6 and max IT time was 100 ms with a scan range of 200–2000 m/z. dd MS2 settings were 15,0000 resolution, AGC target 1e6, max IT time was 100 ms and scan range was 200–2000 m/z.

### N241A/34 Biotinylation and Magnetic Bead Conjugation

One hundred ug Neuromab N241A/34 monoclonal antibody (Antibodies Inc, 75–253) was desalted with a Zeba Spin desalting column, 7 K MWCO, 0.5 ml (Thermo Fisher, 89882) and then biotinylated using a One-Shot biotinylation kit (TriLink Biotechnologies, San Diego, CA, United States). Degree of biotinylation was measured according to One-Shot protocol using a Thermo Fisher NanoDrop spectrophotometer (Waltham, MA, United States) where 2 μL of biotinylated antibody is loaded to read the absorbance at 280 and 354 nm. Absorbances at both wavelengths are input into ChromaLink Biotin Molar Substitution Ratio (MSR) calculator to determine precise degree of biotinylation. An average of six biotin molecules was calculated per one N241A/34 antibody. Biotinylated N241A/34 was combined with streptavidin-coated magnetic beads (M280 Dynabeads, Thermo Fisher, Waltham, MA, United States) at a ratio of 100 μg antibody to 1 mg of beads according to the manufacturers protocol. Final conjugated antibody mixture was stored in 0.1% BSA PBS solution at 4C until use.

### SISCAPA Workflow for Targeted CSF LRRK2 Detection

**FIGURE 1:**
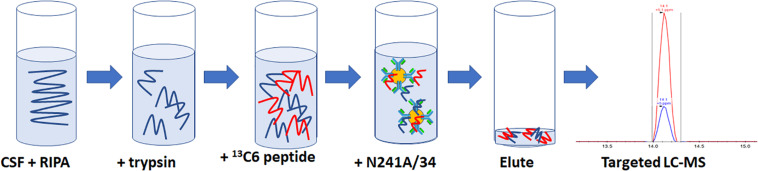
Schematic representation of the SISCAPA workflow used here to detect total LRRK2 levels. CSF is incubated with RIPA buffer and trypsin for 1.5 h at 40C. Samples are put on ice for 5 min and then two pg of heavy labeled 136C15N4 KAEEGDLLVNPDQPR is spiked into the sample. Biotinylated N241A/34 conjugated to M280 streptavidin beads are added to samples to isolate both heavy and light KAEEGDLLVNPDQPR peptides. Beads are washed and eluted. Analysis of light:heavy ratio is done using nanoflow LC and orbitrap mass spectrometry.

For rLRRK2 calibration curve and human CSF experiments, all samples were made up to 1 ml then 100 μl of 10× radioimmunoprecipitation assay (RIPA) buffer (Sigma Aldrich, St. Louis, MA, United States) was added. Twenty micrograms of TPCK-treated trypsin were added to each sample and digested at 40°C for 1.5 h at 1400 RPM shaking on a Thermomixer (Thermo Fisher, Waltham, MA, United States). Following digestion, samples were placed on ice for 5 min then 50 μl of 2 pg/ml (100 fg) heavy synthetic peptide ^13^C12^15^N6 KAEEGDLLVNPDQPR (New England Peptide, Gardner, MA, United States) labeled at both C (K) and (R) termini (*m*/*z* 566.9641+++) was added to each sample. Then, 10 μl of N241A/34 on beads was added to each sample and incubated at 4°C for 1.5 h on an end over end Hula Mixer (Thermo Fisher, Waltham, MA, United States). Beads were then washed using 1 ml of PBS + 0.05% Tween (PBST) on and end over end mixer for 1 min. PBST was removed and then beads were washed twice using 1 ml PBS at 1 min each time. Peptides were eluted off beads with 50 μl of H_2_0 + 0.1% formic acid and 5% acetonitrile (ACN; Figure 1).

### HPLC-Mass Spectrometry Peptide Analysis

A RSLC (Thermo Fisher, Waltham, MA, United States) nanoflow autosampler and HPLC system was used for sample separation. Peptide eluent was injected onto a Thermo C18 Pepmap nano trap column (100 μm i.d. × 20 mm, 5 μm particles) at 20 μl/min for 4.5 min. Peptides were then eluted onto an E800A EasySpray nanoLC column (75× 15 cm, 3 μm particles) nanoLC column at 0.3 μl/min. For all other experiments Q Exactive HFX was operating in parallel reaction monitoring (PRM) mode at 120,000 resolution, AGC target set to 1e^6, maximum injection time (IT) set to 240 ms and isolation window set to 1.0 *m*/*z*. Inclusion list contained both light (*m*/*z* 560.9566+++) and heavy (*m*/*z* 566.9641+++). Samples were analyzed using Skyline 64-bit (University of Washington, MacCoss lab, WA, United States) software and signal was considered detectable if cumulative peak area was >5000 units and contained a minimum of four fragment ions. Most intense fragment ions typically observed were *y5, y6, y7*, and *b6, b7, b8*.

To determine the concentration of each sample (in pg/ml), the light:heavy ratio was taken and then multiplied by two since the internal standard was 2 pg/ml. Finally, we considered the fraction of the entire protein that is being detected, i.e., the peptide is 1/170.4 of the total mass of total LRRK2 (i.e., 1069Da/280,000Da) protein. Therefore, we multiplied the ratio by 170.4 to give us an accurate concentration of the total LRRK2 protein in the sample. This is expressed by:

$$2\,\left(\frac{\text{light}}{\text{heavy}}\right)\times 170.4=\left[\mathrm{LRRK2}\right]\,\left(\frac{p\,g}{m\,l}\right)$$

### Detergent Addition Effects on CSF LRRK2 Levels

Since previous work showing LRRK2 measurements in CSF used a vesicle enrichment step followed by vesicle lysis with detergents, we aimed to determine whether detergent addition (i.e., RIPA) was necessary for the detection of LRRK2 in our CSF samples. Four 2 ml pools of CSF were made and then aliquoted into separated 1 ml tubes. Half the samples (four) had 100 μl 10X RIPA (Sigma Aldrich, St. Louis, MO, United States) while the other half (four) did not. Signals were compared using a paired two-tailed *T*-test.

### CSF Blood Contamination on Total LRRK2 Levels

An 8 ml pool of CSF (BioIVT, Hicksville, NY, United States) was split into 8–1 ml aliquots. Each aliquot had a different volume of fresh whole blood spiked in, ranging from 10 ηl to 10 μl, i.e., 0.001 to 1% v/v whole blood in 1 ml CSF. To be consistent with sample processing at a clinical trial site, samples were frozen at −80°C following whole blood addition then thawed. After thawing the samples, a small volume of the blood-spiked CSF (5 μl) was removed from each aliquot to assess hemoglobin (HgB) levels (Abcam, Cambridge, MA, United States). The rest of the sample was processed using the SISCAPA workflow described here for total LRRK2 detection. To measure Hgb, we used a commercial ELISA kit from Abcam(ab157707). CSF was diluted 1:100 in assay diluent to a final volume of 500 μL. HgB present in the test samples was captured by anti-HgB antibody pre-adsorbed on the surface of microtiter wells after a 20 min incubation under room temperature no shaking. After sample binding, unbound proteins and molecules were washed off, and an enzyme-antibody conjugate was added to the wells and allowed to bind to captured HgB. After 20 min incubation, unbound proteins and molecules were washed and Chromogen substrate solution was then added to catalyze the reaction. After 10 min incubation, stop solution was added. Light intensity, which was proportional to the amount of HgB present, was measured at 435 nm on a SpectraMax plate reader (Molecular Devices, San Jose, CA, United States). HgB concentrations were determined on a standard curve by plotting OD vs concentration using a five-parameter logistical curve-fit. The calibration curve range of this method is 6.25 ηg/ml-200 ηg/ml.

### Michael J Fox Foundation LRRK2 Cohort Consortium CSF

106 CSF samples were collected as part of the MJFF LRRK2 Cohort Consortium and were shipped to Biogen, blinded, in 200 μl aliquots. Five aliquots were pooled (1 ml) in 1.5 ml Eppendorf LoBind tubes and then processed by two separate operators according to the protocol described here including detergent, trypsin and internal standard addition to all samples. Unblinding only took place until after all sample analyses were conducted and raw data was submitted back to MJFF. Age of cohort ranged from 26 to 83 years old. LRRK2+PD+ group included G2019S mutation carriers while the LRRK2-PD+ group had no known mutations. Of the 106 samples analyzed, 105 of samples had evaluable levels of LRRK2.

## Results

### Peptide Mapping of Digested LRRK2 Pull Down With N241A/34

We confirmed that N241A/34 could isolate AEEGDLLVNPDQPR, but also unexpectedly found that it isolated a second high confidence peptide containing a missed cleavage, i.e., KAEEGDLLVNPDQPR (AA 1833–1847). Proteome Discoverer (Thermo Fisher, Waltham, MA, United States) processed the DDa data against the human proteome and showed that KAEEGDLLVNPDQPR gave an 8X higher peak (2.68e8) area than AEEGDLLVNPDQPR (3.27e7; Table 2). In addition, the KAEEGDLLVNPDQPR showed 4 peptide spectral matches (PSMs) compared to 2 for the AEEGDLLVNPDQPR peptide (Table 2).

**TABLE 2:** Peptide mapping results following N241A/34 immunoprecipitation of tryptic digested recombinant LRRK2.

A2	Sequence	# PSMs	# Proteins	# Protein groups	Protein group accessions	Area	Charge	RT [min]	#Missed cleavages
High	KAEEGDLLVNPDQPR	4	1	1	Q5S007	2.681E8	4	11.97	1
High	AEEGDLLVNPDQPR	2	1	1	Q5S007	3.270E7	2	12.48	0

### Recombinant LRRK2 Detection Calibration Curve

**FIGURE 2:**
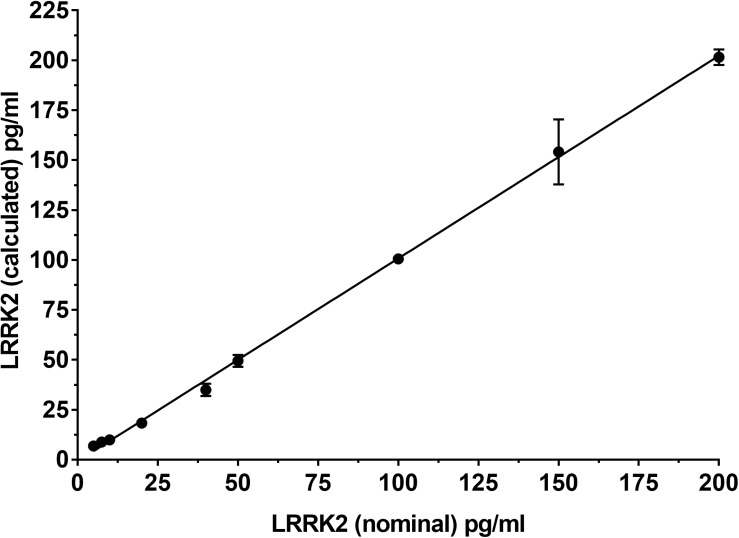
Calibration curve using recombinant LRRK2 from 5–200 pg/ml. Curve includes LQC (7.5 pg/ml), MQC (40 pg/ml) and HQC (150 pg/ml) samples. Curve was run a total of six times and showed acceptable reproducibility.

To determine if the SISCAPA workflow using KAEEGDLLVNPDQPR could give reliable results using rLRRK2 at physiological concentrations, we diluted the protein from 5 to 200 pg/ml in a PBS-BSA (0.1%) solution (Figure 2 and Table 3). We also included 3 QC levels (LCQ = 7.5, MQC = 40, and HQC = 150 pg/ml) to assess assay precision and accuracy. Six calibration curves were generated by two different analysts. Data show that the full SISCAPA process can generate linear calibration curves (*R*^2^ = 0.999) at these concentrations. Bias was calculated for each of the concentration points. Furthermore, endogenous QC (EQC) was included in 4 of the runs (Table 3). Based on these data, we have assigned a 5 pg/ml quantitative limit for this assay. Assay precision was assessed by taking the mean of the LQC, MQC, and HQC across all six individual runs (12.6%) and accuracy was assessed by comparing the determined values of the three QC samples against their nominal values, i.e., the signal bias (10.8%) (Figure 2 and Table 3).

**TABLE 3:** Analytical performance characteristics of the LRRK2 SISCAPA assay using recombinant LRRK2 and an endogenous QC (EQC) CSF sample.

Type	STD	LQC	STD	STD	MQC	STD	STD	HQC	STD	EQC
Pg/ml	5	7.5	10	20	40	50	100	150	200	
Cal1	7.51	9.08	10.09	18.86	32.61	49.73	97.40	154.30	201.41	
Cal2	5.30	9.59	11.10	18.16	31.92	50.40	100.04	184.09	209.08	
Cal3	5.43	6.36	7.54	19.08	36.54	53.18	100.73	142.80	199.04	15.54
Cal4		11.12	9.90	17.20	33.21	44.42	102.87	157.01	200.11	17.77
Cal5	4.28	7.93	9.14	18.91	40.26	51.19	103.24	148.47	198.26	16.53
Cal6	8.17	8.61	11.45	17.55	35.05	47.80	98.76	138.01	201.26	20.05
average	6.14	8.78	9.87	18.29	34.93	49.45	100.51	154.12	201.53	17.47
stdev	1.63	1.60	1.42	0.79	3.11	3.03	2.28	16.29	3.90	1.94
CV%	26.62	18.22	14.37	4.29	8.91	6.13	2.27	10.57	1.93	11.12
Bias%	22.71	17.08	−1.30	−8.54	−12.67	−1.09	0.51	2.74	0.76	

### Human CSF LRRK2 Detection Reproducibility and Effect of Detergent

**FIGURE 3:**
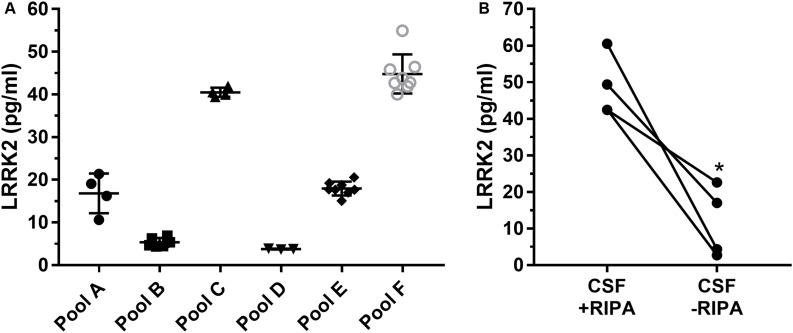
Remnant CSF samples were pooled (A–F) and then split into separate 1 ml aliquots. Samples were then processed in parallel to determine run to run variability **(A)**. Overall variability was limited to 12% across pools A–F. Addition of radioimmunoprecipitationassay (RIPA) buffer which contains detergents greatly enhances signal of LRRK2 in CSF **(B)**. ^∗^*p* < 0.05.

Since the SICAPA workflow appeared to provide consistent data using rLRRK2 (Figure 2 and Table 3), we moved to using human CSF (Figure 3). In order to confirm that the assay could be performed reliably in this context we made 6 different pools of CSF (A-F) ranging from 3 to 8 ml (Figure 3A). Each pool was then divided into separate 1 ml aliquots. Each pool was fully processed in parallel. Different pools were run at different times by a total of four separate analysts. LRRK2 levels within each pool ranged from 4 to 54 pg/ml. Individual %CV from each pool was between 3% (Pool B,D) to 27% (Pool A). The mean CV% across all six different experiments was 12% (Figure 3A).

To determine whether or not detergent addition (RIPA buffer) had an effect on LRRK2 detection, presumably through vesicle disruption during proteolysis, we compared CSF with and without 100 μl RIPA in each sample (Figure 3B). Data show that CSF containing RIPA had 48.7 ± 8.5 pg/ml LRRK2 while CSF samples without RIPA had 11. 7 ± 9.7 pg/ml therefore detergent contributed to a >four-fold increase in LRRK2 levels as analyzed by a paired two-tail *T*-test, *p* = 0.0165 (Figure 3B).

### Effect of Blood Contamination on CSF LRRK2 Levels

**FIGURE 4:**
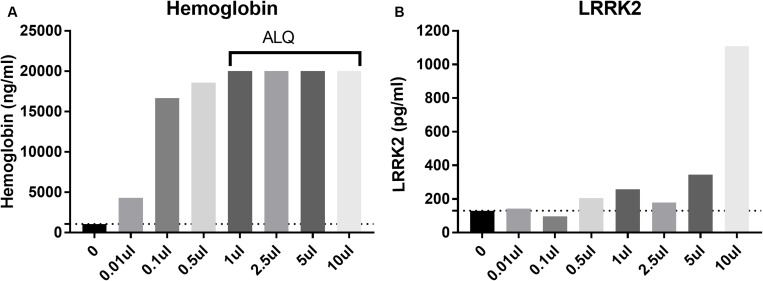
Spiking in whole blood into CSF dramatically increases measured hemoglobin levels **(A)** with all samples more than 1 ul (0.01% v/v whole blood spiked in) having levels above the limit of quantification (ALQ) of the assay. LRRK2 levels in those same samples did not increase significantly until 5 μl of whole blood was spiked into 1 ml [i.e. when blood made up 0.5% of the entire sample by volume; **(B)**].

CSF blood contamination caused by traumatic lumbar puncture can have a negative impact on CSF measures particularly when protein analyte is highly expressed in blood such as alpha synuclein (aSYN). Since LRRK2 is expressed in circulating white blood cells including monocytes, macrophages and leukocytes (Hakimi et al., 2011), we wanted to determine to what extent blood contamination would affect LRRK2 protein levels. We performed both LRRK2 analysis and hemoglobin analysis following whole blood addition to 1 ml CSF. Data show that LRRK2 levels are not greatly affected by whole blood addition to CSF until >5 μl/1 ml of CSF (i.e., 0.5% v/v), when CSF is visibly discolored by blood (Figure 4B). At this level, hemoglobin levels exceed their assay quantitation limit (Figure 4A).

### Analysis of CSF LRRK2 Levels From the LRRK2 Cohort Consortium

**FIGURE 5:**
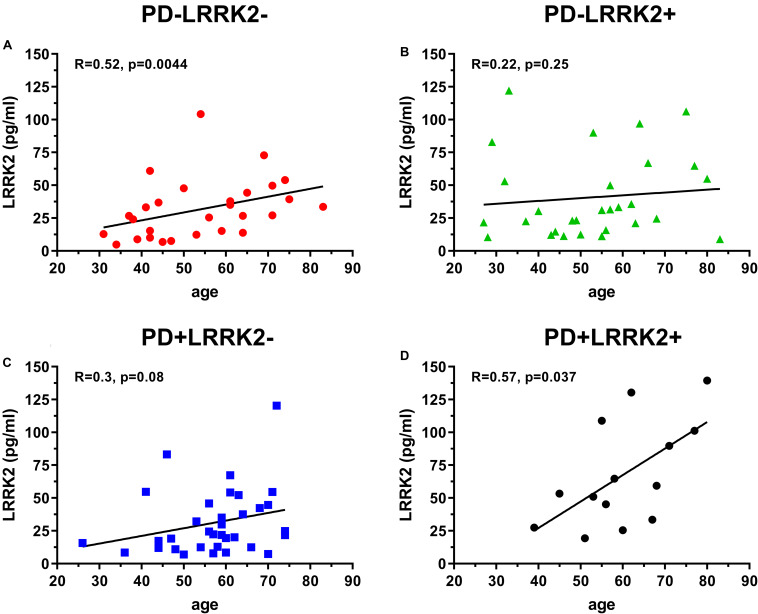
106 CSF samples were analyzed using the LRRK2 SISCAPA assay. Of the 106 samples analyzed, 105 had measurable LRRK2 levels. Overall correlation between CSF LRRK2 levels and age in the LRRK2 Cohort Consortium samples using Spearman’s rank correlation in the PD-LRRK2- **(A)**, PD-LRRK2+ **(B)**, PD+LRRK2– **(C)**, and PD_LRRK2+ **(D)** groups.

**FIGURE 6:**
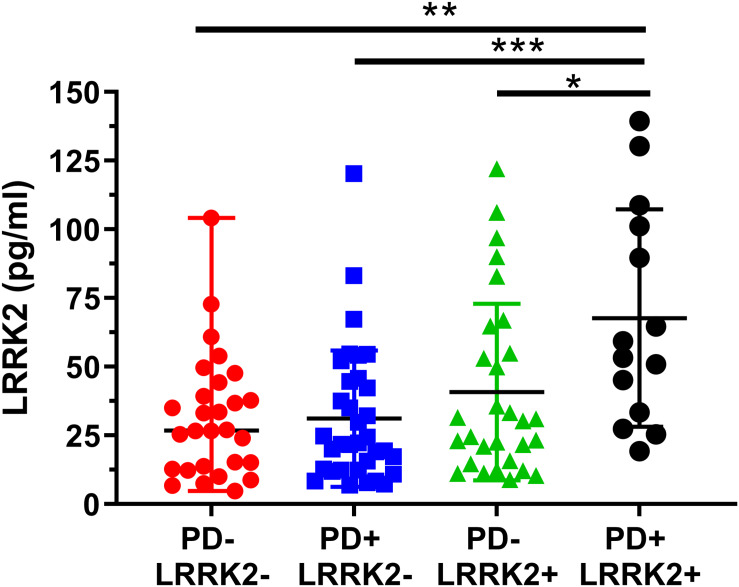
Analysis of mean CSF LRRK2 levels in the LRRK2 Cohort Consortium samples shows that LRRK2 was significantly higher in the PD+ LRRK2+ group compared to all other groups. No other groups were significantly different from each other. ^∗^*p* < 0.05, ^∗∗^*p* < 0.01, ^∗∗∗^*p* < 0.001.

One hundred and six (106) human CSF samples were received from the MJFF LRRK2 Cohort Consortium in a blinded fashion. Of the 106 samples, 105 had evaluable levels of LRRK2 (Figures 5, 6). Mean LRRK2 concentrations ± SD for healthy controls (*n* = 28) was 31.7 ± 22.7 pg/ml (range = 5–104 pg/ml). For sporadic (non LRRK2 carriers) PD (*n* = 34) it was 31.1 ± 24.8 pg/ml (range = 7–120 pg/ml). For LRRK2+PD- non manifesting carriers (*n* = 29) it was 40.8 ± 32.1 pg/ml (range = 9–122 pg/ml). For LRRK2+PD+ patients (*n* = 14) mean levels were 67.8 ± 39.6 pg/ml (range = 19–139 pg/ml). The effect of age was measured by plotting total LRRK2 levels against age for each of the 4 groups separately (Figures 5A–D). Data show that LRRK2 gradually, yet significantly increased with age in the PD-LRRK2- (healthy control; *R* = 0.52, *p* = 0.0044; Figure 5A) and the PD+LRRK2+ groups (*R* = 0.57, *p* = 0.037) but not in the PD-LRRK2+ (Figure 5B) or PD+LRRK2- groups (Figure 5D).

We then analyzed group differences in total LRRK2 levels. A one-way ANOVA showed a significant effect between the four groups (F3, 101 = 2.04, *p* = 0.0007; Figure 6). A Bonferroni post hoc analysis showed that the LRRK2+ PD group had significantly higher CSF LRRK2 compared to healthy controls (*p* = 0.0013), idiopathic PD (*p* = 0.0007), and compared to non-manifesting LRRK2 carriers (*p* = 0.029).

We then applied an ANCOVA model and included the term for Cohort and adjusted for age as a continuous variable, and gender as a categorical variable. The adjusted mean is the expected mean value of the outcome calculated from the model with the value of age being the average age across cohort, and with an equally weighted gender covariate value (50% being in each gender) in the model, for each cohort (Table 4). A Bonferroni post hoc analysis for multiple comparisons with a 95% confidence interval adjustment is shown on Table 4.

**TABLE 4:** ANCOVA Statistical analysis of CSF LRRK2 levels following age and gender adjustment.

group	Adjusted LRRK2 mean value	95% Confidence Interval	*p*-values (a)
PD-LRRK2−	32.28	(21.74, 42.83)	
PD-LRRK2+	42.57	(32.20, 52.94)	
PD+LRRK2−	30.70	(20.97, 40.42)	
PD+LRRK2+	65.10	(50.20, 80.00)	*p* < 0.01 (vs PD+LRRK2−); *p* < 0.01 (vs PD-LRRK2−)

*Bonferroni post hoc analysis for multiple comparisons with a 95% confidence interval adjustment is shown.*

## Discussion

The current work describes a novel quantitative methodology for reliably detecting total LRRK2 levels in 1 ml of human CSF. The method does not require an exosome isolation step and uses a commercially available antibody (N241A/34) within the SISCAPA workflow which is scalable and amenable to higher throughput analyses. We demonstrated that the assay meets our basic fit for purpose qualification criteria such as dilutional linearity across a physiological dynamic range and acceptable precision (12.6%) and accuracy (10.8%). Furthermore, we show that blood contamination in CSF does not pose a serious analytical challenge compared to other analytes that are more highly expressed in blood such as aSYN. Finally, using this methodology we showed that PD patients harboring the G2019S LRRK2 mutation have significantly higher CSF LRRK2 levels compared to healthy individuals, sporadic PD patients, and non-manifesting LRRK2 carriers.

The search for a sensitive and high throughput assay to detect LRRK2 and/or pLRRK2 levels in CSF has been a challenge for both academic investigators and industry teams seeking to advance clinical stage LRRK2 therapeutic programs. Our internal work suggested that a number of high quality antibody reagents could be used to develop ultrasensitive immunoassays (e.g., Quanterix Simoa, Sinuglex Erenna, MSD S-plex) that could measure low pg/ml concentrations (Biogen Internal; Padmanabhan et al., 2020). Although those assays performed well in tissues such as rodent and primate brain and human PBMCs, they could not reliably detect LRRK2 in human CSF with or without exosome isolation. One hypothesis for this was that the native conformation of LRRK2 protein in CSF (whether folding, dimerization or aggregation) could limit epitope accessibility, while WB analysis would provide sufficient denaturation to allow binding to antibodies such as MJFF2 or N241A/34. Indeed, several reports from the West lab have demonstrated LRRK2 detection by WB analysis of vesicle enriched CSF (Fraser et al., 2013, 2016; Wang et al., 2017). Since WB analysis is not amenable to clinical trials, we sought an alternative CSF LRRK2 detection approach. The SISCAPA workflow is ideal for proteins requiring strong denaturation since it relies on total proteolysis to generate peptides which are targeted by a capture antibody. This peptide level enrichment greatly reduces sample complexity and increases mass spectrometer signal by removing interferents and other matrix effects. Another advantage of the SISCAPA workflow is high confidence signal specificity (encoded by high resolution mass spectrometry), which can be difficult to prove in standard immunoassays.

The assay was enabled by the discovery that N241A/34 performed well as an anti-peptide antibody and could isolate the sensitive missed cleavage peptide KAEEGDLLVNPDQPR which was confirmed only to exist in mammalian LRRK2 protein (Standard Protein BLAST, NIH). The use of a missed cleavage peptide as a surrogate for LRRK2 protein detection was initially a concern because it was not clear how well we could control its generation compared to the fully cleaved peptide (i.e., AEEGDLLVNPDQPR). Tryptic miscleavage occurs for many reasons such as adjacent cleavage sites, nearby glutamic or asparatic acid residues or phosphorylation (Šlechtová et al., 2015). In the current context, sequential K residues (AA 1832–1833) are considered a classic missed cleavage pattern which greatly reduces digestion efficiency. Once KAEEGDLLVNPDQPR is generated, trypsin does not efficiently cleave the N terminal K because in this position (far N term) it is no longer a good trypsin substrate (Yen et al., 2006). Furthermore, because of the proximity of several negatively charged glutamic acids (E), trypsin would be even less efficient cleaving the N terminal K. Šlechtová et al. (2015) characterized the efficiency of digestion of different K and R containing peptides, including missed cleavage peptides. They found digestion efficiency of a peptide containing a single K to be similar to a peptide containing KK (32 vs 26% efficiency, respectively). However, they also found that the missed cleavage product is subsequently digested at a 6,000 X slower rate than a single K residue in the middle of a peptide sequence (Šlechtová et al., 2015). In other words, AEEGDLLVNPDQPR and KAEEGDLLVNPDQPR are generated at approximately the same rate, but once KAEEGDLLVNPDQPR is generated, the likelihood that it is cleaved further to AEEGDLLVNPDQPR remains low. We can use these digestion kinetics to our advantage and apply a rapid digestion protocol which stably generates the KAEEGDLLVNPDQPR peptide. A number of reproducibility experiments both with recombinant LRRK2 and endogenous LRRK2 (Figures 2, 3A) gave us confidence that the missed cleavage peptide could be reliably generated. Parallel processing of the same CSF gave reproducible results and if the generation of KAEEGDLLVNPDQPR was stochastic in nature, then processing the same sample would generate more variable results. Our data show that across a number of runs, a CV of ∼12% was observed across 4 different operators analyzing the same CSF sample (Figure 3A). This variability also takes into account immunoprecipitation efficiency and LC-MS performance, suggesting that digestion is highly reproducible.

Using this assay we showed that blood contamination did not cause a significant pre-analytical issue for LRRK2 detection as it does for aSYN (Mollenhauer et al., 2017), consistent with a previous study which showed that CSF samples with high HgB levels had normal pS1292 LRRK2 (Wang et al., 2017). This is likely because LRRK2 (unlike aSYN) is not expressed in erythrocytes (Hakimi et al., 2011) which make up approximately half of whole blood volume. Similar to previous reports (Fraser et al., 2013; Wang et al., 2017), our human CSF data did show a wide range of concentrations between subjects which is likely due to true biological variability and not blood contamination arising from traumatic lumbar puncture. We observed from 4 to 55 pg/ml in our reproducibility study and levels between 5 and 139 pg/ml in the MJFF LRRK2 Cohort Consortium samples. The significance of these differences is not clear, and it is also not known if these levels would correspond to total LRRK2 levels in the brain, which is not testable in the absence of matched ante-mortem CSF and post-mortem brain samples. It is conceivable that elevated LRRK2 protein would translate into greater LRRK2 kinase activity which is thought to be involved in the pathogenicity of LRRK2 mutations (Greggio et al., 2006; Henry et al., 2015; Alessi and Sammler, 2018; Di Maio et al., 2018) and data from Skibinski et al. (2014) suggests that higher LRRK2 protein expression itself is an important driver of elevated kinase activity and pathological inclusion body formation. Conversely, multiple reports have shown that reductions in LRRK2 kinase activity may also reduce total LRRK2 levels pointing toward a tight relationship between total LRRK2 levels and its activity state (Lin et al., 2009; Herzig et al., 2011; Skibinski et al., 2014).

The main finding of the current study is that LRRK2+ PD patients have elevated CSF LRRK2 levels compared to healthy controls, sporadic PD patients or LRRK2+ non-manifesting carriers. Even after adjusting for age and gender (Table 4), LRRK2 levels in CSF were still statistically higher in the PD+LRRK2+ group compared to the non LRRK2+ groups. One hypothesis that could explain this observation is that the LRRK2+ PD+ group has higher levels of cytosolic LRRK2 localization and enhanced secretion which is controlled through 14-3-3 interactions (Nichols et al., 2010; Fraser et al., 2013). Previous preclinical studies using cell models have shown that LRRK2 kinase activity modulates the interaction between LRRK2 and 14-3-3, and pathogenic LRRK2 mutations cause LRRK2 to accumulate in the cytoplasm (Greggio et al., 2006; Nichols et al., 2010; Di Maio et al., 2018). Another study described how increased LRRK2-14-3-3 interactions result in enhanced extracellular release through exosomes (Fraser et al., 2013). Although that study failed to show that G2019S mutations were sufficient to enhance LRRK2+ exosome release, they did show that kinase inhibition using a small molecule inhibitor could reduce LRRK2+ exosome release (Fraser et al., 2013). Therefore, it is plausible that G2019S carriers with PD have enhanced cytosolic LRRK2 levels and trafficking into exosomes which would be reflected in higher CSF LRRK2. Although the current assay did not strictly target exosome contents in CSF, trypsin and detergent were added (RIPA buffer used contains deoxycholic acid and NP40) to samples which may be enough to disrupt vesicles storing LRRK2 protein and our data show that when samples were processed without detergent, this resulted in significantly lower LRRK2 levels (Figure 3B). Therefore, it is likely that the LRRK2 detected in the current study reflects all CSF LRRK2 content, including vesicular and non-vesicular derived protein. The data provided here also suggest the LRRK2 mutation alone is not sufficient to increase CSF LRRK2 since the PD- LRRK2+ group did not have significantly elevated levels compared to the healthy controls or the sporadic PD group, though a trend was observed. This suggests a potential interaction with LRRK2 and another process that occurs in PD. One hypothesis is that mutated LRRK2-aSYN interactions promotes LRRK2 expression and/or mistargeting within the cell and subsequent release into interstitial fluid / CSF. A previous study showed that reducing aSYN in G2019S rat neurons could reduce total LRRK2 levels (Skibinski et al., 2014). Perhaps our observation reflects an interaction between mutated LRRK2 and aggregated aSYN that promotes LRRK2 expression which is not seen in the PD- LRRK2+group. Similar mechanisms have been proposed by Eguchi et al. (2018), where they demonstrated that lysosomal overload stress induced the recruitment of endogenous LRRK2 onto lysosomal membranes via Rab7L1 (Rab29). The complexity of PD pathogenesis and the incomplete penetrance of the LRRK2 mutations clearly indicate that multiple factors contribute to LRRK2’s role in disease. Multiple lysosomal / endosomal genes have been linked to PD (Nalls et al., 2019; Ebanks et al., 2020) and it is likely that even in absence of known pathogenic mutations in the cohorts included in the present study, polygenic risk factors (see Iwaki et al., 2020) and/or environmental factors act synergistically with the mutant LRRK2 to drive pathogenesis. These hypotheses are speculative and additional preclinical and postmortem CNS tissue work, together with a more detailed genetic analysis of disease-manifest LRRK2 mutation carriers (compared to carriers without disease), will be needed to support these claims. The development of the assay described here provides a critical tool for future experiments which will detail whether within subject longitudinal CSF LRRK2 is stable enough to be useful in clinical trials aimed at modifying LRRK2 levels over time.

In summary, the assay described here provides a reliable means to measure total LRRK2 levels in human CSF which could be used to support interventional clinical trials where LRRK2 is targeted. Future iterations of the assay should include techniques to capture kinase activity (i.e., multiplexing with pRab10, for example) and should be optimized to reduce sample volume requirements. Additional steps can also be taken to automate this assay to improve throughput. Importantly we showed that in a set of 105 CSF samples that the LRRK2+PD+ group had roughly 2× higher CSF LRRK2 levels compared to other groups. Although the precise reasoning for this increase has yet to be elucidated, future studies can use this quantitative methodology to probe the relationship between disease progression and longitudinal CSF LRRK2 levels.

## Data Availability Statement

The datasets generated for this study can be found in the LRRK2 Cohort Consortium database.

## Ethics Statement

Ethical review and approval was not required for the de-identified sample analysis in accordance with the local legislation and institutional requirements. The patients/participants provided their written informed consent to participate in this study.

## Author Contributions

OM developed the assay described here and executed the experiments. SC and AE performed the experiments. MY performed statistical analyses on the LRRK2 Cohort Consortium CSF the data. WH and DG conceptualized experiments and gave guidance throughout the assay development process.

## Conflict of Interest

All authors are employed by Biogen, Cambridge, MA, United States. Biogen has obtained a non-exclusive patent license for SISCAPA assay research use from SISCAPA ASSAY Technologies, Inc. for the assay.

## Footnotes

1 www.clinicaltrials.gov
